# Revealing platelet-related subtypes and prognostic signature in pancreatic adenocarcinoma

**DOI:** 10.1186/s12920-023-01530-x

**Published:** 2023-05-17

**Authors:** Jian-Gang Zhao, Yu-Jie Li, Yong Wu, Ke Zhang, Lin-Jia Peng, Hao Chen

**Affiliations:** 1 Department of Oncology, Shaoxing Central Hospital, Shaoxing, 312030 China; 2 Department of Oncology, Shanghai Medical College, Fudan University, Shanghai, 200032 China; 3 Department of Integrative Oncology, Fudan University Shanghai Cancer Center, 270 Dong An Road, Shanghai, 200032 China; 4 Department of Oncology, The second affiliated Hospital of Anhui University of Traditional Chinese Medicine, Hefei, 230061 China

**Keywords:** Pancreatic adenocarcinoma, Platelet, Molecular classification, Prognostic signature, risk score

## Abstract

**Background:**

Pancreatic adenocarcinoma (PDAC) is a malignant tumor with high heterogeneity and poor prognosis. In this study, we sought to identify the value of platelet-related genes in prognosis and heterogeneity of PDAC through multiple transcriptomic methods.

**Methods:**

Based on datasets from Gene Expression Omnibus and The Cancer Genome Atlas (TCGA), platelet-related genes were screened out, and the TCGA cohort (n = 171) was identified into two subtypes by unsupervised clustering. The platelet-related risk score model (PLRScore) was constructed by univariate Cox and LASSO regression, and the predictive ability was evaluated by Kaplan-Meier test and time-dependent receiver operating characteristic (ROC) curves. The results were validated in two other external validation sets, ICGC-CA (n = 140) and GSE62452 (n = 66). Furthermore, predictive nomogram containing clinical characteristics and PLRScore was established. In addition, we determined the possible correlation between PLRScore and immune infiltration and response of immunotherapy. Finally, we analyzed the heterogeneity of our signature in various types of cells using single-cell analysis.

**Results:**

Platelet-related subtypes that have significant difference of overall survival and immune states (*p* < 0.05) were identified. PLRScore model based on four-gene signature (CEP55, LAMA3, CA12, SCN8A) was constructed to predict patient prognosis. The AUCs of training cohort were 0.697, 0.687 and 0.675 for 1-, 3-and 5-year, respectively. Further evaluation of the validation cohorts yielded similar results. In addition, PLRScore was associated with immune cell infiltration and immune checkpoint expression, and had promising ability to predict response to immunotherapy of PDAC.

**Conclusions:**

In this study, the platelet-related subtypes were identified and the four-gene signature was constructed and validated. It may provide new insights into the therapeutic decision-making and molecular targets of PDAC.

## Introduction

Pancreatic adenocarcinoma (PDAC) is one of the malignant tumors with high morbidity and mortality [1]. Extensive intra-and inter-tumor heterogeneity has been revealed to be an important cause of poor prognosis, so it is crucial to improve the accuracy of clinical treatment decisions based on the molecular characteristics of PDAC [2]. In recent years, molecular subtypes have guided the development and treatment strategies for a wide range of malignancies [3]. Various molecular subtypes have been identified in PDAC, including the Collisson’s [4] and Moffitt’s [5] subtypes. Molecular subtyping strategy enabled patients to predict the optimal treatment strategy before treatment, thus improving the overall survival of patients.

The experimental evidences showed that platelets not only inhibit blood loss and promote wound healing, but also play an active role in tumor growth, tumor cell extravasation and tumor metastasis [6]. Platelets and their derived growth factors play an essential role in protecting and promoting tumor metastasis, such as assisting tumor evasion from host immune surveillance [7]. In addition, increased platelets in cancer patients were associated with poorer survival [8]. Recently, platelet transcriptomics has emerged as a highly sensitive method for characterizing malignant tumors, where the combination of large data cohorts and machine-learning algorithms enables precise feature selection and potential prognostication [9]. However, the study of platelet-related genes in molecular subtypes of pancreatic cancer has not been reported.

In conclusion, platelets are closely related to the growth and progression of cancer. In this study, platelet-related genes were collected and characterized by multi-omics analysis. Then we identified the platelet-related subtypes in PDAC and analyzed the biological characteristics of the subtypes. Then we screened the prognostic factors to construct a prognostic risk model that could differentiate the prognosis of PDAC. Furthermore, the correlation between risk score and clinical features or immune infiltration of PDAC was analyzed.

## Materials and methods

**Fig. 1:**
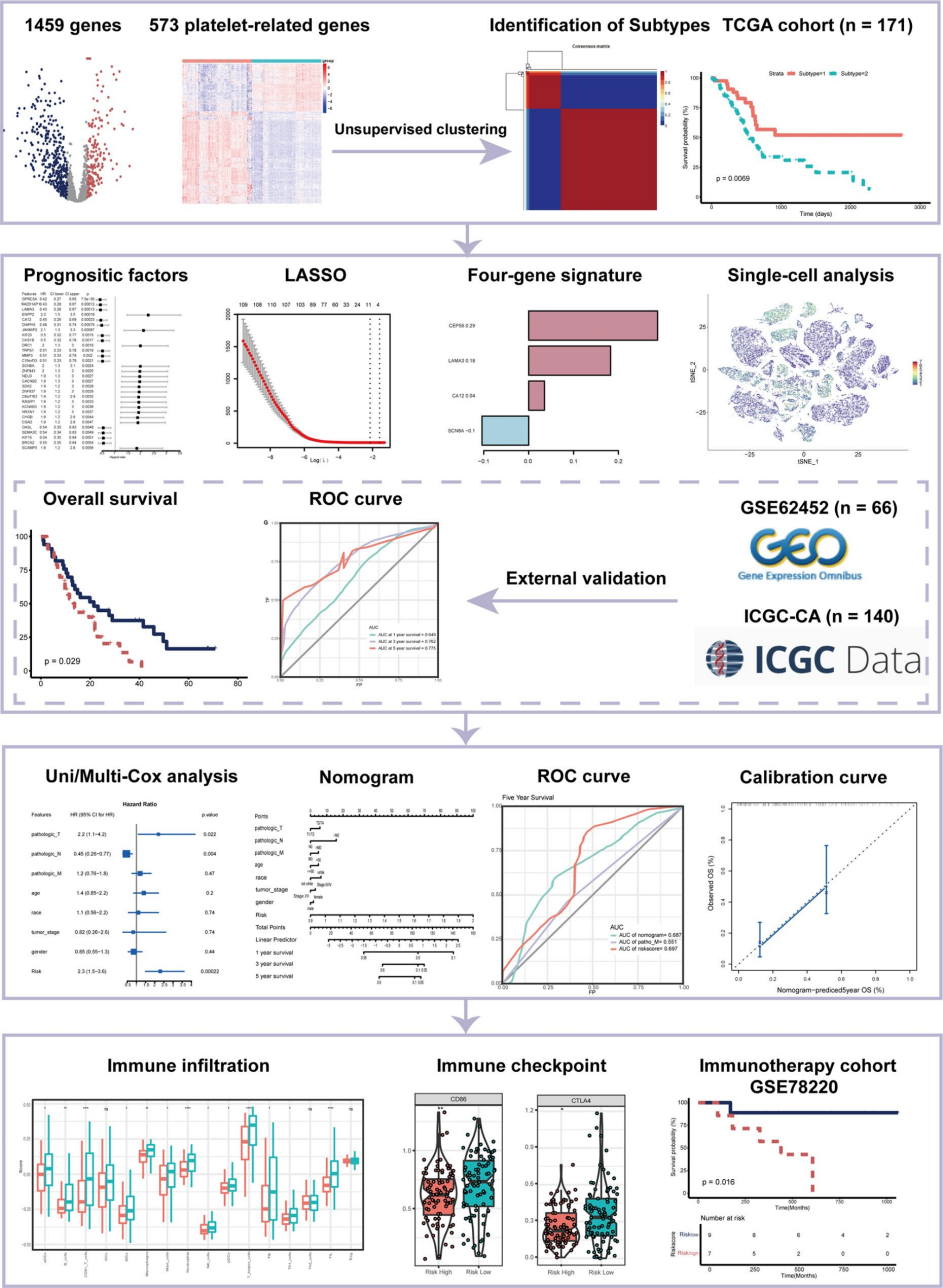
Workflow of the current study

A schematic presentation of the research procedure is shown in Fig. 1.

### Data availability and preprocessing multi-omics

To identify the platelet related genes, the gene expression data were downloaded from The Genotype-Tissue Expression (GTEx, https://gtexportal.org/) and Gene Expression Omnibus (GEO, https://www.ncbi.nlm.nih.gov/geo/) dataset GSE160252 (https://www.ncbi.nlm.nih.gov/geo/query/acc.cgi?acc=GSE160252). The RNA-seq raw read count, fragments per kilobase million (FPKM) and the related clinical information from patients with PDAC were extracted from The Cancer Genome Atlas (TCGA, https://portal.gdc.cancer.gov/projects/TCGA-PAAD/) database which were used for the construction of our signature. Only samples with survival information were screened for follow-up analysis (TCGA-PAAD, n = 171). For validation of the prognostic model, the clinical follow-up and gene expression were obtained from GSE62452 (n = 66, https://www.ncbi.nlm.nih.gov/geo/query/acc.cgi?acc=GSE62452) and International Cancer Genome Consortium (ICGC-CA, n = 140, https://dcc.icgc.org/). In addition, the Expression profiling and therapeutic information of GSE78220 (https://www.ncbi.nlm.nih.gov/geo/query/acc.cgi?acc=GSE78220) was downloaded for use in immunotherapy-related analysis.

### Identification of platelet-related genes

The differentially expressed platelet-associated genes were selected (*p* value < 0.05, |log2FoldChang| > 1) according to the GSE160252, and 1459 genes were obtained. Then, based on the differential expression genes between PDAC and normal pancreatic tissue, 573 differential platelet-related genes (PRGs) were selected from 1,459 genes using DEseq2 package (FDR < 0.01, |log2FoldChang| > log2(3)). The PRGs in the study were performed for Gene Ontology (GO) and Kyoto Encyclopedia of Genes and Genomes (KEGG) pathway [10] enrichment analysis by using the ‘clusterProfiler’ R package.

### Identification of platelet-related subtypes in PDAC

Using the ‘Non-negative matrix factorization (NMF)’ R package (rank = 2: 6), patients were consensus clustered into two subtypes based on PRGs, and the Kaplan-Meier (K-M) method with log-rank test was performed to compare overall survival (OS) differences between the subgroups. In order to study the differences between two subtypes in platelet-related pathways, gene set variation analysis (GSVA) algorithm was used to analyze the angiogenesis and epithelial to mesenchymal transition (EMT)-related pathway scores. To analyze the differences of immune infiltration status between the two subtypes, CIBERSORT and ESTIMATE algorithms were carried out using R. Then, immune profile differences between subtypes were estimated by Wilcoxon test.

### Construction and validation of Prognostic Model based on PRGs

Firstly, the PRGs were tested using the univariate Cox analysis to determine the prognostic value. Subsequently, based on the candidate genes with *p* < 0.05 in the univariate Cox analysis, a least absolute shrinkage and selection operator (LASSO) regression model was constructed by “glmnet” package. Finally, the hazard ratios (HR) and regression coefficients for each gene used in the construction of the final prognostic signature. The risk score was calculated using the following formula:

$$riskscore={e}^{{\sum }_{k=1}^{n}{coef}_{k}{expression}_{k}}$$

where e refers to the natural constant, expression_k_ is the expression of the kth selected gene, and coef_k_ is its regression coefficient. The survival analysis was implemented to compare the OS of the two subgroups. The time-dependent receiver operating characteristic (ROC) analysis at 1, 3, and 5 years of prognostic value was used to assess discrimination of the model in predicting OS of PDAC using the R package ‘survivalROC’. External validation was conducted in ICGA-CA and GSE62452 cohorts, respectively.

### Assessment of prognostic factors and construction of a predictive nomogram

To assess whether the risk score was able to combined with clinical factors as an independent prognostic factor, uni- and multi-Cox regressions were used to verify the prognostic role of them. Then, a nomogram was established using R package ‘rms’ based on risk score and clinical factors. The predictive effect of the nomogram was validated by ROC and calibration curve.

### Correlations between risk score and immunocyte infiltration

CIBERSORT algorithm and single sample gene set enrichment analysis (ssGSEA) was applied in analyzing the differences of immune infiltration status between high- and low-risk groups. The boxplot was also used to show the difference in the expression of immune checkpoint between high- and low-risk groups.

### Single cell analysis

Expression matrix for single cell transcriptome analysis and clinical characteristics of PDAC were obtained from CRA001160 in the Genome Sequence Archive (GSA, https://ngdc.cncb.ac.cn/gsa/browse/CRA001160) database. To demonstrate the heterogeneity of our signature in the single-cell dimension, the expression of platelet-related signature in each cell was calculated and compared among different cell types. ‘Seurat’ R package was used to generate t-distributed Stochastic Neighbor Embedding (t-SNE) plot for cell type visualization. Comparisons among all cell types were conducted through Kruskal-Wallis test.

### Statistical methods

Comparisons between two groups were conducted through the Wilcoxon test. The survival curve was generated by Kaplan-Meier method and the log-rank test was used to determine the significance of the difference. All statistical analyses were performed using the R programming language (Version 4.1.0). A difference of *p* < 0.05 indicated statistical significance unless specified otherwise.

## Results

### Selection and Functional Enrichment of PRGs

**Fig. 2:**
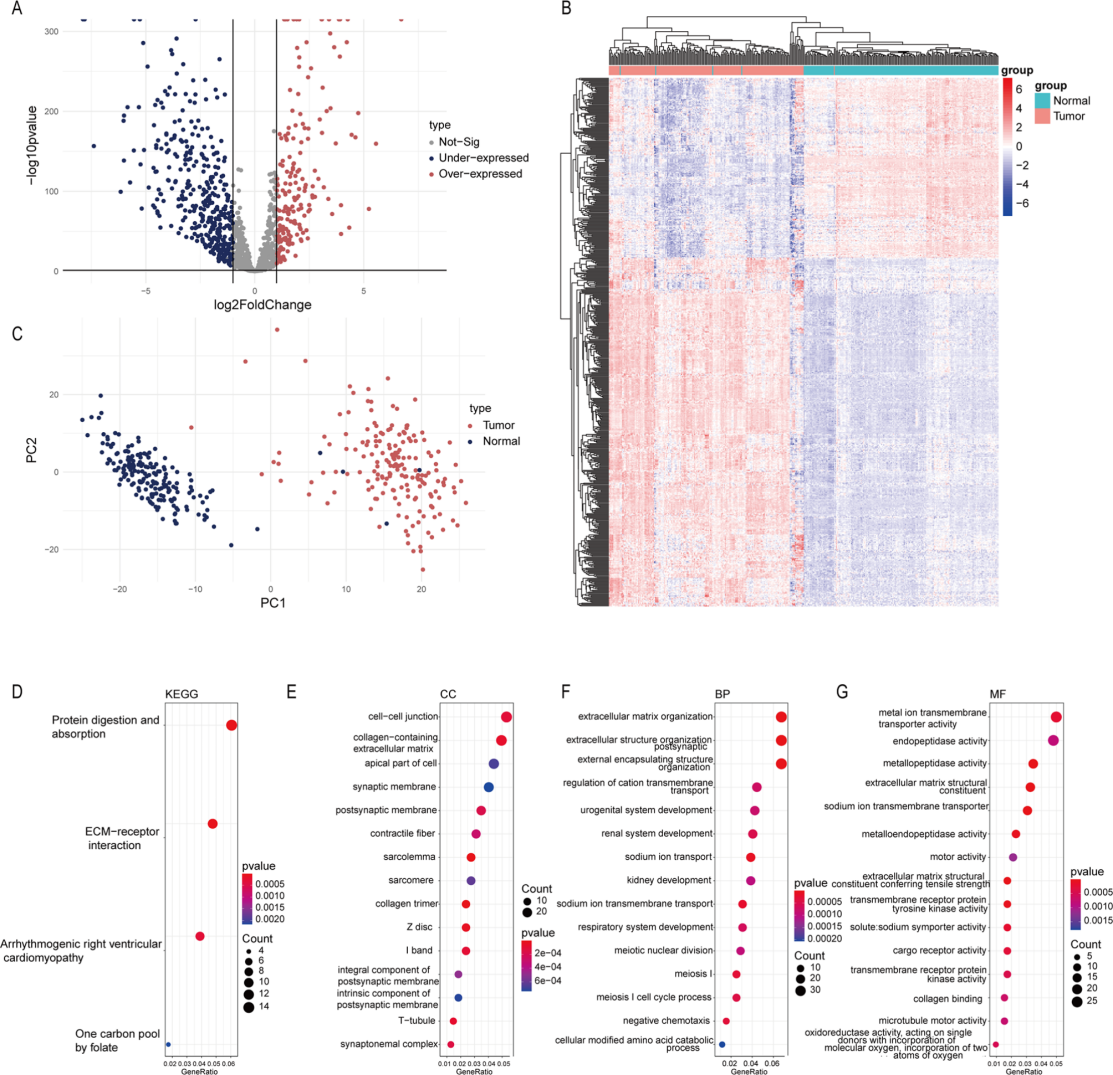
Identification of platelet-related genes in pancreatic adenocarcinoma (PDAC). (**a**) The differentially expressed platelet-associated genes were selected from GSE160252 and showed in volcano plot. (**b**) The heatmap showed that 573 platelet-related genes (PRGs) were selected by differential analysis between PDAC and normal tissues. (**c**) Principal component analysis (PCA) showed a significant distribution difference of PRGs between PDAC and normal tissues. The enrichment of PRGs by Kyoto Encyclopedia of Genes and Genomes (d) and Gene Ontology (**e**-**g**)

1459 platelet-associated factors were obtained from the GSE160252 dataset, and 573 PRGs were selected by differential analysis between PDAC and normal tissues. The results were visualized by volcano plot (Fig. 2A) and heatmap (Fig. 2B). Principal component analysis (PCA) showed a significant distribution difference of PRGs between PDAC and normal tissues (Fig. 2C). In order to explore the potential biological functions and pathways of PRGs, we performed functional enrichment analysis. Interestingly, the KEGG analysis showed that PRGs were highly enriched in several biological processes, including protein digestion and absorption, ECM − receptor interaction, arrhythmogenic right ventricular cardioamyopathy and one carbon pool by folate (Fig. 2D). And the PRGs were significantly enriched in the pathways associated with intercellular communication and the promotion of fibrogenesis in the tumor microenvironment in GO analysis (Fig. 2E-G).

### Different characteristics of platelet-related subtypes

**Fig. 3:**
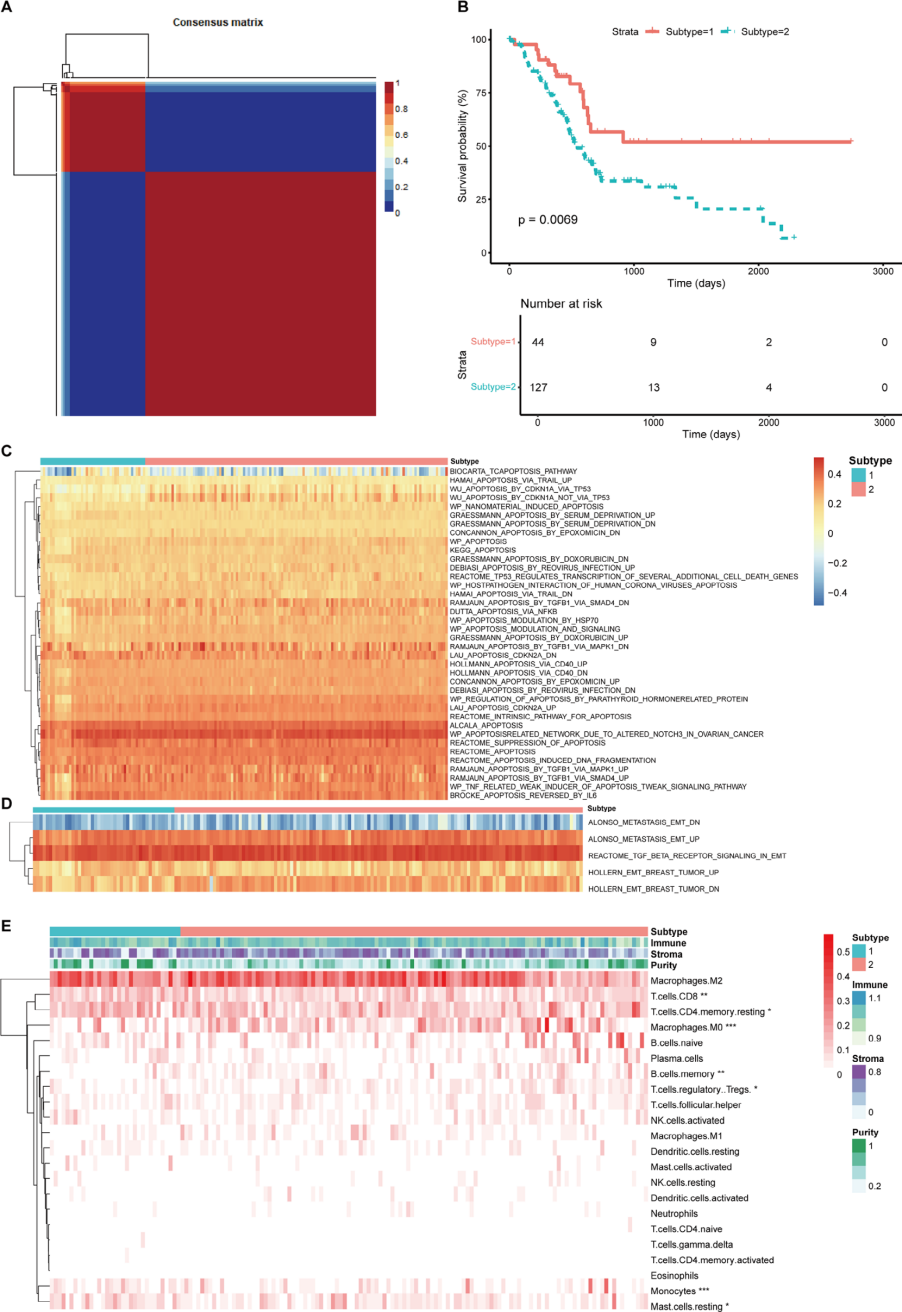
Identification of platelet-related subtypes in the TCGA cohort (n = 171). (**a**) The consensus matrix of the platelet-related genes. (**b**) Overall survival (OS) of patients in subtype1 and subtype2 (p < 0.05). The heatmap showed GSVA scores of the angiogenic (**c**) and EMT-related pathways (**d**). (**e**) The heatmap showed immune infiltration of the two subtypes by CIBERSORT and ESTIMATE algorithms. * represents *p* < 0.05, ** represents *p* < 0.01, *** represents *p* < 0.01

In order to reveal the heterogeneity of platelet-related genes in PDAC, 171 patients were divided into two subtypes according to the expression profile of PRGs (Fig. 3A). And there was significantly difference of OS between the two subtypes (Fig. 3B). Subtype2 (n = 127) had an inferior prognosis than subtype1 (n = 44) which promoted us to find the difference of biological processes and immunocyte infiltration between two platelet-related subtypes. The heatmap showed GSVA scores of the angiogenic (Fig. 3C) and EMT-related pathways (Fig. 3D). And there were significant differences in apoptosis-associated pathways between the two subtypes. The immune landscape of the two subtypes was further studied (Fig. 3E). The subtype1 had higher immune score, stromal score, ESTIMATE score and lower tumor purity than the subtype2. In addition, the CIBERSORT algorithm was used to analyze 22 different immune cell types in the two subtypes. In the subtype1, CD8 T cells, CD4 memory resting T cells, monocytes and rest mast cells were up-regulated, and macrophages (M0), Regulatory T cells (Tregs) and memory B cells were down-regulated significantly (*p* < 0.05).

### Construction of platelet-related risk score (PLRScore) model

**Fig. 4:**
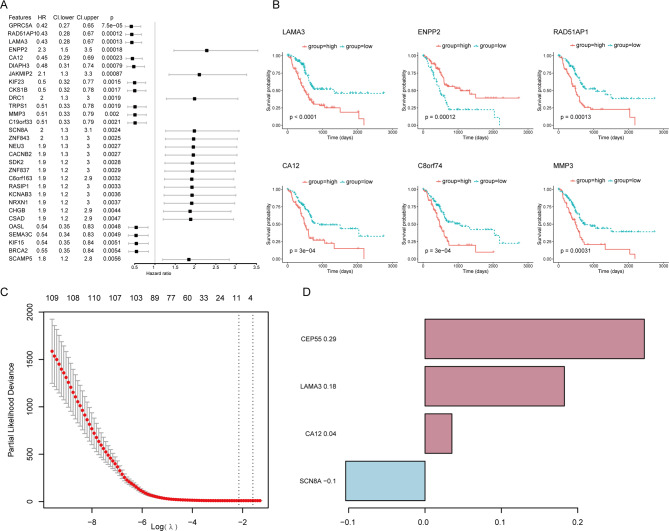
Construction of the four-gene signature. (**a**) Forest plot of univariate Cox regression analysis showed that top 30 PRGs were associated with prognosis. (**b**) P values differed most significantly among the top 6 genes in the survival curve. (**c**) Selection of the tuning parameter (lambda) in the least absolute shrinkage and selection operator (LASSO) regression by 10-fold cross-validation based on minimum criteria for OS. (**d**) The coefficient of four genes of the signature

Platelet-related subtypes could distinguish patients with different prognosis, but the number of genes is too large for clinical application. To build a simple scoring model, univariate Cox regression showed that 112 PRGs were potential prognostic factors, and the results were partly shown by forest plot (Fig. 4A). And the patients were divided into two groups based on the median expression of these potential prognostic factors, significant survival differences between the two groups were identified (the six most significant were shown, Fig. 4B). To establish PLRScore in patients with PDAC in the TCGA dataset, these 112 PRGs were included in the LASSO regression (Fig. 4C). An optimal 4 genes (CEP55, LAMA3, CA12, SCN8A) signature and coefficient of each were identified (Fig. 4D).

### Analysis of prognostic efficiency of PLRScore model in training set and validation sets

**Fig. 5:**
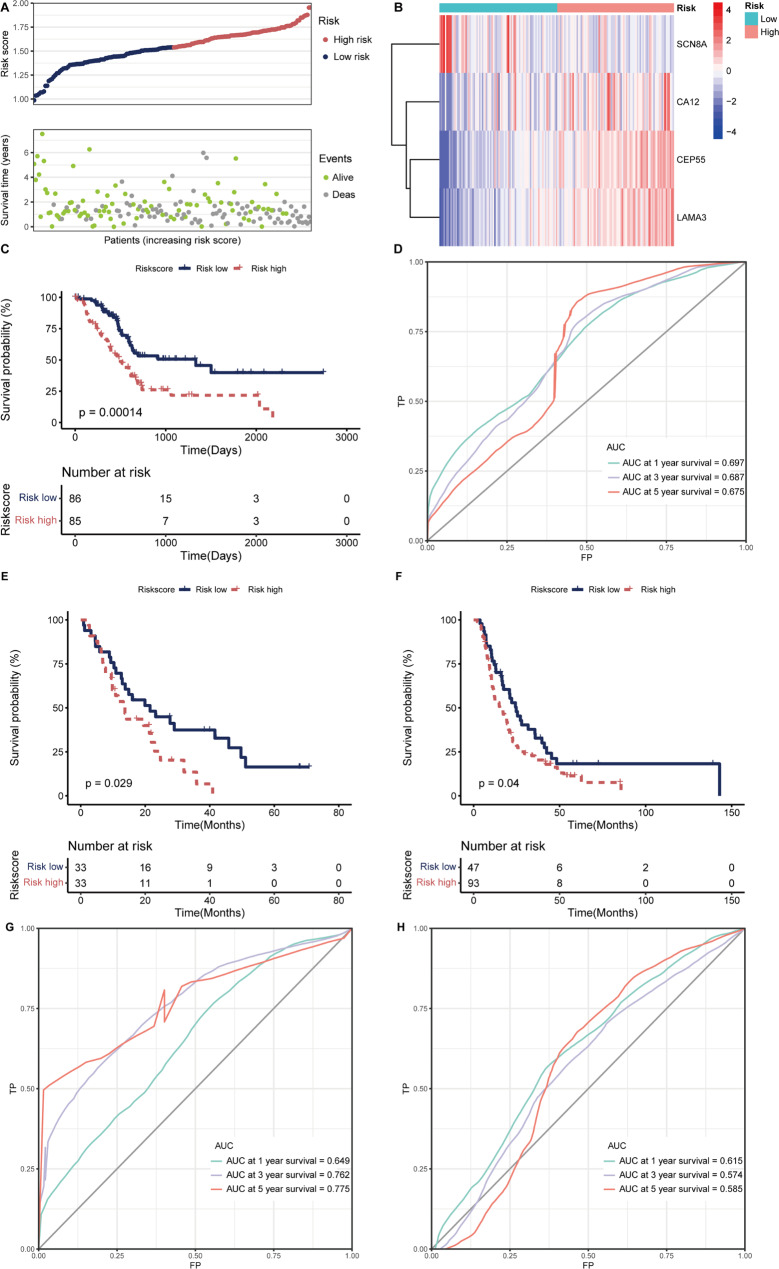
Construction and validation of the platelet-related risk score (PLRScore) model. Distributions of risk scores, survival status (**a**) and expression of 4 platelet-related genes (**b**) in the TCGA cohort (n = 171). (**c**) Kaplan-Meier (K-M) curves for the OS of patients in the high- and low-risk groups in the TCGA cohort. (**d**) The receiver operating characteristic (ROC) curve analyses of the prognostic PLRScore in the TCGA cohort. The OS of patients in the high- and low-risk groups in the GSE62452 cohort (**e**, n = 66) and ICGC cohort (**f**, n = 140). The ROC curves of the prognostic PLRScore in the GSE62452 cohort (**g**) and ICGC cohort (**h**)

According to the median PLRScore, patients were divided into PLRScore-high (n = 85) and PLRScore-low (n = 86). The increased PLRScore was accompanied by gradually decreasing survival time and increased mortality (Fig. 5A). And the expression level of 4 genes signature was presented in form of heatmap (Fig. 5B). Kaplan-Meier survival analysis showed that OS of the PLRScore-high was significantly shorter than that of the PLRScore-low (Fig. 5C). The AUCs at 1, 3 and 5 years were 0.697,0.687 and 0.675, respectively. (Fig. 5D). To verify the stability of the model constructed from the TCGA dataset, patients in the GSE62452 were also assigned to PLRScore-high and low groups relative to the median risk score which was calculated using the same formula as the training set (Fig. 5E). To avoid the extreme population allocation, patients in the ICGC dataset were assigned to PLRScore-high and low groups relative to the lower quartile risk score (Fig. 5F). There were significant differences in survival between the two groups of validation sets (*p* < 0.05). The AUCs of the PLRScore at 1, 3 and 5 years in the GEO cohort were 0.649, 0.762 and 0.775 (Fig. 5G) and in the ICGC cohort were 0.615, 0.574 and 0.585 (Fig. 5H), which proved the generality of our model.

### The independence of the PLRScore in predicting OS in PDAC

**Fig. 6:**
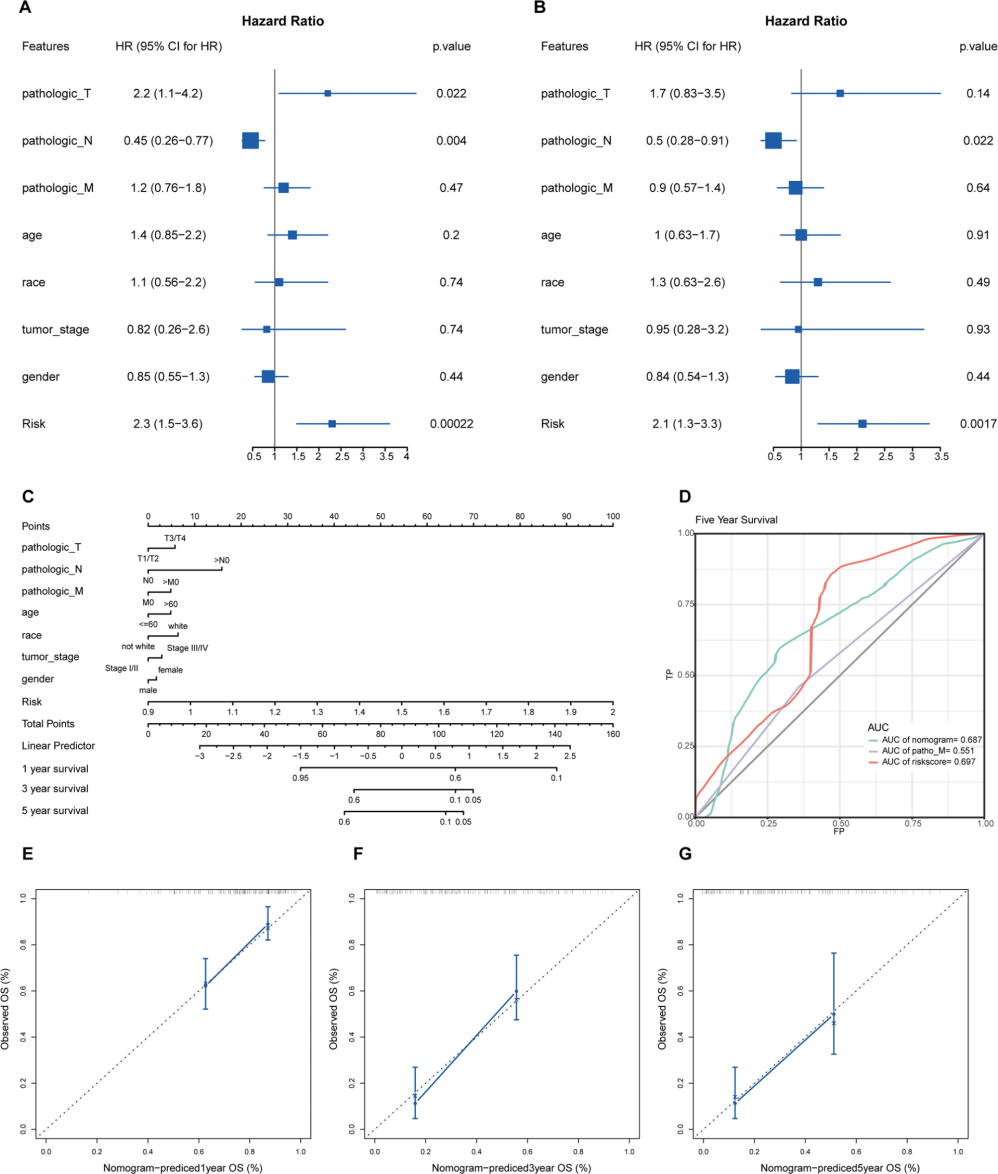
Development of a prognostic nomogram based on clinical factors and PLRScore. Forest plot of univariate (**a**) and multivariate (**b**) Cox regression analysis showed that PLRScore was an independent factor for prognosis. (**c**) Nomogram based on risk score, age, race, gender, tumor stage and TNM stage. The ROC curves (**d**) and calibration curves (**e**-**g**) for the nomogram

To evaluate whether PLRScore was an independent prognostic indicator for OS, univariate and multivariate Cox regression analyses were performed. In the TCGA dataset, both univariate Cox analysis and multivariate Cox analysis showed a significant correlation between N stage and PLRScore and overall survival (Fig. 6A, B). In order to establish a predictive tool for clinical use, a prognostic nomogram was developed based on pathological TNM stage, age, race, tumor stage, gender and PLRScore (Fig. 6C). The AUC of the nomogram was 0.687 (Fig. 6D). The calibration curves showed that the nomogram has prediction consistency (Fig. 6E-G). These results indicated that the PLRScore can be applied as an independent prognostic factor combined with clinical indicators.

### The role of PLRScore in predicting the efficacy of immunotherapy

**Fig. 7:**
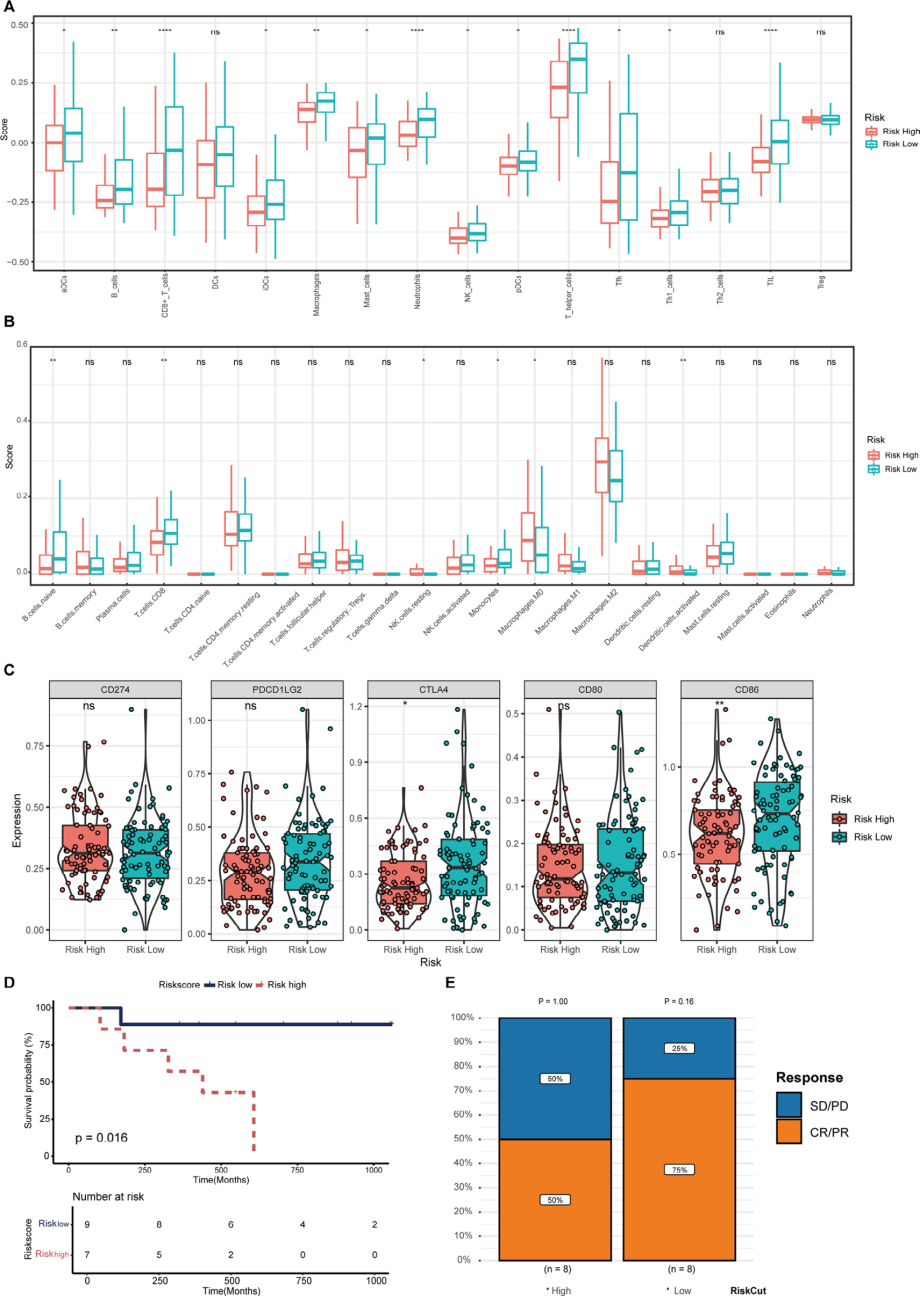
The immune landscape between high- and low-risk groups. Single sample gene set enrichment analysis (**a**) and CIBERSORT algorithm (**b**) were applied in analyzing the differences of immune infiltration status between high- and low-risk groups. (**c**) The violin plot was used to show the difference in the expression of immune checkpoint between high- and low-risk groups. (**d**) K-M curve for the OS of patients in the high- and low-risk groups in the GSE78220 cohort. (**e**) The response of immunotherapy between the high- and low-risk groups. * represents *p* < 0.05, ** represents *p* < 0.01, *** represents *p* < 0.01, ns represents no significant difference

To further explore the correlation between immunity and PLRScore, enrichment scores of different risk groups and related functions or pathways were used in the ssGSEA analysis. As expected, the PLRScore-low group scored higher on the immune landscape, including CD8^+^ T cells, neutrophils, T helper cells, TILs, B cells, macrophages, NK cells, mast cells, activated dendritic cells (aDCs), iDCs, pDSs, Tfh and Th1 cells (Fig. 7A). CIBERSORT analysis showed differences of cell infiltration in tumor microenvironment between PLRScore-high and low groups. In the PLRScore-low group, infiltration scores of CD8^+^ T cells, naïve B cells and monocytes were significantly augmented, along with decreased infiltration score of resting NK cells, M0 macrophages and aDCs (Fig. 7B). We also used boxplot to show the difference in the expression of immune checkpoints between the two groups. The differences in the expression of cytotoxic T-lymphocyte-associated protein 4 (CTLA-4) and CD86 between PLRScore-high and low groups were significant (Fig. 7C). To explore whether the PLRScore could predict responses of immunotherapy, a significant correlation between PLRScore and OS was observed in the GSE78220 data from 16 melanoma samples (Fig. 7D, E). The results demonstrated the ability of our PLRScore to predict the prognosis of patients undergoing immunotherapy.

### Heterogeneity of the four-gene signature among cell populations in PDAC

**Fig. 8:**
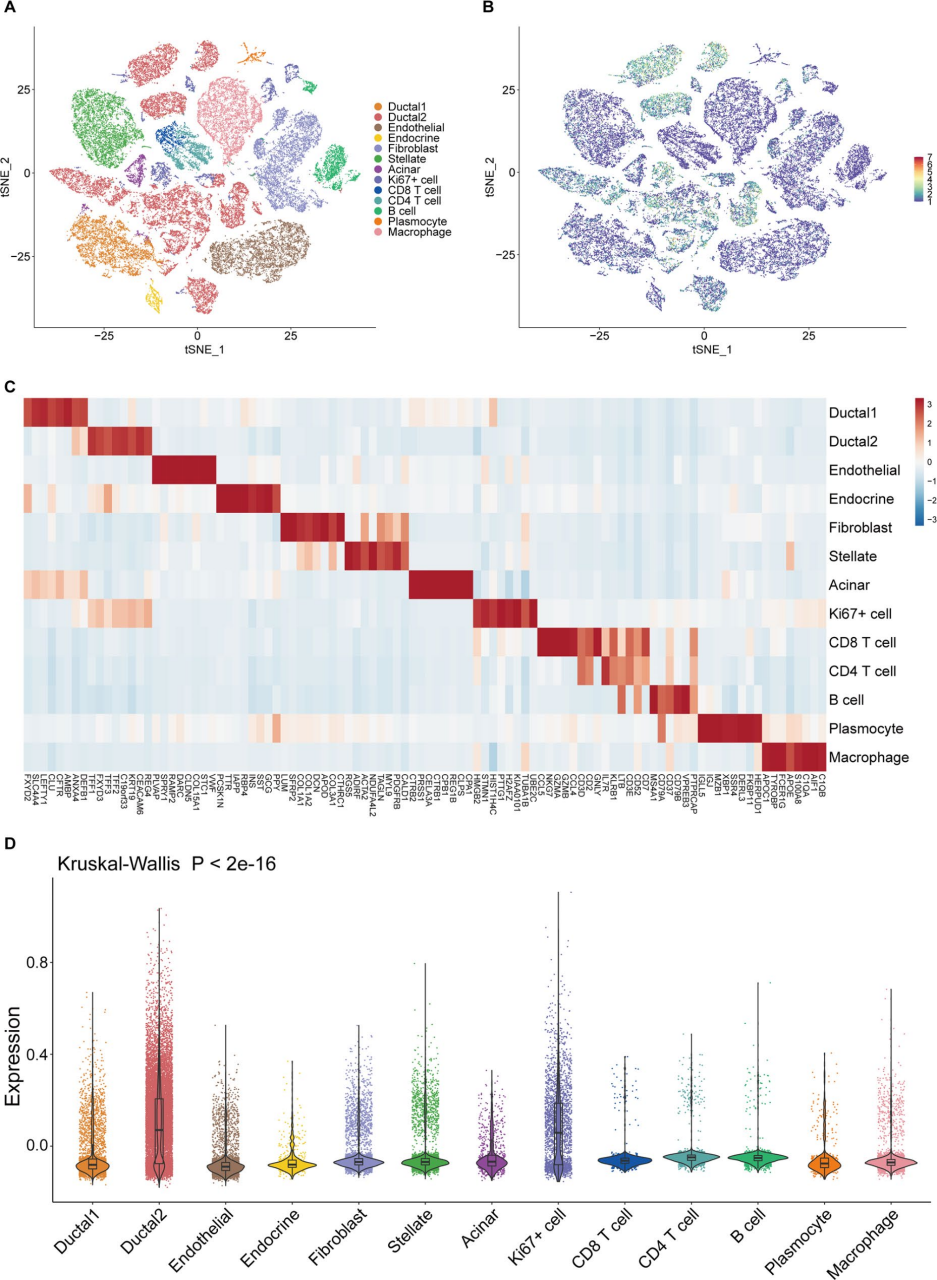
Single-cell analysis of heterogeneity of the four-gene signature in PDAC (**a**) The t-distributed stochastic neighbor embedding (t-SNE) plot demonstrated main cell types in PDAC. (**b**) The expression of four-gene signature among the cell types. (**c**) Heatmap showing expression levels of specific markers in each cell type. (**d**) Kruskal-Wallis test showed the expression of four genes in different cell types were significantly different

Based on Peng’s et.al [10] study, we performed a principal component analysis and unsupervised clustering of the variable expression genes in all cells and identified 33 clusters, and used well-established marker genes to classify the 33 clusters into 13 cell populations including type 1 ductal cell, type 2 ductal cell, Ki67^+^ cell, CD8^+^ T cell, CD4^+^ T cell, B cells, macrophages, endothelial cells, endocrine cells, fibroblasts, plasmocytes, stellate cells and acinar cells (Fig. 8A). We further investigated the expression of the four-gene signature in single-cell populations, which was significantly increased in both type 2 ductal cells and Ki67^+^ cells (Fig. 8B). The heatmap showed the representative marker genes for each cell cluster (Fig. 8C). And Kruskal-Wallis test showed the heterogeneity of our signature at the single-cell level (Fig. 8D).

## Discussion

Our previous studies have found that some hemostatic parameters are independent prognostic factors for pancreatic cancer, and based on these hemostatic parameters we have developed a scoring system for predicting survival in patients with advanced pancreatic cancer [11]. Recent studies have shown that platelets participate in tumor growth and metastasis by stimulating tumor cell proliferation and promoting tumor angiogenesis through the release of various cytokines and chemokines [12]. In this study, we aimed to find relationship between platelet and prognosis of patients with PDAC at the level of the transcriptome. And two different prognostic platelet-related subtypes were identified and a prognostic model was developed, which was validated by two independent external cohorts.

We identified two subtypes of pancreatic cancer with different prognosis by screening platelet-related genes, and attempted to speculate the reasons for their different prognosis. Subtype2, the one with poor prognosis, was upregulated in macrophages, Tregs and memory B cells and downregulated in CD8^+^ T cells, CD4^+^ memory resting T cells, monocytes and rest mast cells. Treg cells are a subtype of immune cells that work to suppress excessive immune activation [13]. A recent study has shown that Tregs mediate immunosuppression through adenosine in tumor microenvironment with abnormal metabolism [14]. In an independent cohort, M0 macrophages were found to be a poor prognostic factor for hepatocellular carcinoma, and angiogenic genes were highly enriched in the M0-high group [15]. These results suggested that the prognosis of platelet-associated subtypes may be related to the tumor immune microenvironment.

To promote the clinical transformation of platelet-associated subtypes, we developed a four-gene signature that predict pancreatic cancer. CEP55 (Centrosome protein 55) was considered essential for cell cycle processes, and there were increasing evidences that up-regulation of CEP55 was involved in the development and progression of various malignancies, including liver cancer [16], bladder cancer [17], anaplastic thyroid cancer [18], non-small-cell lung cancer [19, 20] and colorectal cancer [21]. And overexpression of CEP55 was associated with genomic instability [22, 23]. A number of recent bioinformatics studies have found that LAMA3 (Laminin subunit alpha3) was a poor prognostic factor for PDAC [24–26]. In addition, experiments have verified that LAMA3 showed an increasing trend in the occurrence and development of PDAC [27]. In Gao’s et.al [28] risk prediction model, CA12 (Carbonic anhydrase 12) showed a higher expression level in the high-risk group, and similar to our findings, was a risk factor for prognosis in pancreatic cancer. In contrast, another study found that low CA12 expression was significantly associated with poor overall survival [29]. SCN8A (Sodium Voltage-Gated Channel Alpha Subunit 8), a member of the gene family encoding sodium channel α subunit [30], was found to be highly expressed in colorectal cancer tissues and positively correlated with lymph node metastasis of colorectal cancer [31]. And among epithelial ovarian cancer samples, lower SCN8A expression was associated with improved overall survival [32]. However, SCN8A was defined in our signature as a protective factor for PDAC.

Immune checkpoint inhibitors, targeting CTLA-4 and the programmed cell death protein-1 (PD-1)/programmed cell death ligand-1 (PD-L1) pathways have shown remarkable potential in malignant tumors [33]. CTLA4 has been shown to inhibit T cell activation by capturing and internalizing CD80 and CD86 in antigen presenting cells [34]. In this study, the expression of CTLA4 and CD86 were higher in PLRScore-low group which suggested that the response of immunotherapy may differ between the two groups. And as we expected, the prognosis of patients with immunotherapy was significantly different between PLRScore-low and high groups. Their immune response rates were different, even though there was no significance due to the limited sample size.

Our study had the following limitations: First of all, although we combined bulk and single-cell sequencing data to establish and validate our model, further in vivo and in vitro mechanism exploration may still provide additional information and validation of platelet-related markers for improved treatment strategy in PDAC. Second, the sample size of the external independent immunotherapy cohort we applied was too small, and further clinical trials are also entailed. Finally, we merely discussed the role of platelet in a transcriptomic aspect, the epigenetic process of platelets which is essential in cardiovascular research [35], and the important role of miRNA in platelets [36] needs further discussion.

## Conclusion

This study identified a new platelet-related classification of PDAC, and constructed a risk score model, PLRScore. Our platelet-related subtypes provided insights into the mechanisms associated with immunosuppression and poor prognosis in PDAC. This study described heterogeneity in PLRScore at bulk and single-cell transcriptomic levels. Our four-gene signature, particularly those that have not been studied in PDAC, may provide new insights into the development of more effective biomarkers to predict prognosis and immunotherapy response in patients with PDAC.

## Data Availability

The datasets generated and/or analysed during the current study are available in the TCGA, https://portal.gdc.cancer.gov/projects/TCGA-PAAD/; GTEx, https://gtexportal.org/; GSE160252, https://www.ncbi.nlm.nih.gov/geo/query/acc.cgi?acc=GSE160252; GSE62452, https://www.ncbi.nlm.nih.gov/geo/query/acc.cgi?acc=GSE62452; GSE78220, https://www.ncbi.nlm.nih.gov/geo/query/acc.cgi?acc=GSE78220; ICGC, https://dcc.icgc.org/; GSA, https://ngdc.cncb.ac.cn/gsa/browse/CRA001160.

## List of abbreviations

PDAC: Pancreatic adenocarcinoma
GTEx: Genotype-Tissue Expression
GEO: Gene Expression Omnibus
ICGC: International Cancer Genome Consortium
TCGA: The Cancer Genome Atlas
FPKM: Fragments per kilobase million
PRGs: Platelet-related genes
GO: Gene Ontology
KEGG: Kyoto Encyclopedia of Genes and Genomes
BP: Biological process
CC: Cellular component
MF: Molecular function
NMF: Non-negative matrix factorization
K-M: Kaplan-Meier
OS: Overall survival
GSVA: Gene set variation analysis
EMT: Epithelial to mesenchymal transition
LASSO: Least absolute shrinkage and selection operator
HR: Hazard ratios
ROC: Receiver operating characteristic
GSA: Genome Sequence Archive
t-SNE: t-distributed Stochastic Neighbor Embedding
PCA: Principal component analysis
